# Trichloracetic Acid in Pyorrhea

**Published:** 1893-11

**Authors:** 


ARTICLE V.

### TRICHLORACETIC ACID IN PYORRHEA.

Dr. F. T. Van Woert.—Some time ago—just how long I cannot remember—my attention was called to trichloracetic acid by a short article in one of the journals, that came from the pen of our esteemed colleague, Dr. Peirce, of Philadelphia; and later I began using the drug in connection with pyrozone, the combination of which has given me results in the treatment of pyorrhea so remarkably satisfactory that, like the old-time exalter, I feel like shouting "Glory!" There has been nothing presented to the profession during my practice that promises so much. A patient, a lady about thirty-five years of age, has been in my hands for treatment the last year and a half, and I could not see that I had made the least advance towards success. The disease was apparent throughout the whole mouth; but the lower anterior teeth were the ones that I had about decided to remove, and as a last effort to save them I began treating with the trichloracetic acid and pyrozone. At the end of ten days, during which practically only two treatments had been given, I had the satisfaction of finding the parts in a healthy condition—not the least sign of pus, and apparently a perfect union between the soft tissues and the tooth, which seems to me almost marvelous. What the ultimate outcome will be I can only guess, as the whole thing is so entirely new. I should be very glad if any of the gentlemen present who have used these drugs, and particularly in the treatment of pyorrhea, would relate their experience; and those who have not I would advise to do so.

Dr. Edward C. Kirk.—I think it was a little over a year ago that my attention was called to the use of trichloracetic acid through a note in one of the medical journals; it was there recommended for the removal of vascular growths upon the mucous membrane. I sent for an ounce sample, and made some experiments with it in the treatment of the vascular tumors of the pulp and hypertrophy of the gum-festoon so frequently seen in connection with caries of the sixth-year molar in children, in which the cavity is filled up with the growth, and the margin greatly inflamed and presenting a very unhealthy appearance. I used for the removal of these growths a very strong solution of about ninety per cent., by an application of the solution on cotton placed around a piece of orange-wood. With this I could wipe out the growth a layer at a time, and there was no hemorrhage or pain during the removal. My next experiment with it was in cases of pyorrhea, and I am glad to add my testimony to that of Dr. Van Woert as to its exceedingly remarkable action in these cases. I have tried nearly everything that has been recommended for the treatment of pyorrhea, and I have had perhaps the average success. A lady who had been treated for a chronic condition of pyorrhea for many years fell into my hands as a patient, and her lower incisors were very loose. They were all dancing up and down in pus, and it looked as though there was no alternative but to extract them. I said, here is a case that I can experiment with, and if I should not be able to save the teeth the patient will be no worse off than before treatment. So I proceeded to remove the deposits, using applications of trichloracetic acid for the purpose of softening the concretions upon the roots. I went vigorously to work with the idea of thoroughly removing every particle of deposit. The second visit, which occured about a week afterward, showed that there was no discharge of pus from four of the teeth; two showed a little pus-discharge. The gum-margin of these two teeth, instead of adhering closely to the teeth with a sharp, thin margin, was thickened and inverted, looking as though the teeth were stuck in a batch of soft dough. I made the second and third treatment, using the trichloracetic acid and pyrozone, as before, in connection with the use of scalers. About the third visit, I found that the gum around three of the lower incisors had completely changed in appearance, and, instead of the inverted character which it had formerly presented, was normal in appearance, showing a fine, sharp line of attachment, and was in a perfectly healthy condition. I then examined further, and found that the other teeth which did not have this close vital attachment of the gum had some small deposit or tartar. I persevered until I had removed absolutely everything in the shape of calcareous deposit from the roots, and I have the six teeth now in healthy condition. The remainder of the teeth in the mouth have been without treatment, and are still typical cases of pyorrhea. But I am so much impressed with the results in this case that, as I have said to one or two of my colleagues in Philadelphia, I am almost ready to go on record for the statement that where we do not cure pyorrhea, it means that we have not removed every particle of deposit from the root. I am inclined to this because of the very closely adhering quality of the tartar, for though I thought I had completely removed it I found still more; but afterward, when I had completely removed it, complete restoration followed. I want, however, to try it further before I make so dogmatic a statement.

Dr. R. Ottolengui.—I would like to ask Dr. Kirk whether he thinks a restoration of that kind could be made where the pulp had been removed, or whether he would only expect that to occur where the pulp was alive?

Dr. Kirk.—I never think about pyorrhea in that sense. I simply try experiments and note results. I regard pyorrhea as one of the disorders that we have no business to speculate about. On general principles, I should think the chances against a pulpless tooth; but that is merely a speculation, and I feel, in view of our lack of exact knowledge on the subject that I have no right to make that kind of a guess in relation to any case of pyorrhea.

Dr. Van Woert.—In the case which I recited, one of the teeth was dead, and there was no apparent difference in the action of the trichloracetic acid. It acts as an escharotic, and while there is no restoration of tissue, there is adhesion, and the parts look healthy. In the case that I speak of, I have united the teeth by bridging, and they are doing very nicely.

Dr. Ottolengui.—I would say just a few more words on that question which I asked. I have a patient who had a pyorrhea pocket around a tooth in his mouth; the gum had receded. Recently he came in complaining that he had been in agony and had had a sleepless night. His theory was that I must destroy the pulp: the whole tooth had become so sensitive that he could not put hot or cold water in his mouth without having it ache. I asked him to allow me to experiment with it, and I treated the pocket with pyrozone. He came again in the afternoon and said that he had had some relief, and I gave him a second treatment. He was in the next morning, and I gave him acetanilid to take during the night; I treated it again that morning with pyrozone, and the following morning. I saw nothing of him for a week. In the mean time I became a convert to the trichloracetic acid, and determined to try it on him; but when he came in, I found that the pocket had closed up, and here within a week and a half it has reattached itself and the pain is gone. The point is, that it is possible with these new remedies to get good results.

Dr. Kirk.—Since I have gotten the result that I have noted with trichloracetic acid, and since I have found that it is possible to get a certain kind of restoration—I mean a confirmation of the attachment that is already there, not necessarily reproduction of lost tissue—I have reorganized my system of scaling teeth. I thought I had been doing it pretty well, but since I have been using the treatment noted I have been very much more careful to assure myself that every particle of tartar is removed. The point I wish to make is that after every part seems smooth, and I have considered that the root was clean, I have afterward found that it was not so by adopting the method ot packing the pocket with cotton for a moment for the purpose of distending it, and then bathing the pocket with trichloracetic acid, which coagulates the surface so that no effusion takes place. You can then momentarily get a good look at the root-surface, when you will see any little particles of tartar which remain. You might build a telegraph line from here to San Francisco, and have it all completed with the exception of one inch, but a message could not be passed over the wire; and in like manner this disorder cannot be cured until the last granulate of tartar is removed and the root is thoroughly clean. I have used trichloracetic acid as strong as ninety per cent., simply as an escharotic; but in pyorrhea I have used it only of ten per cent. strength. Trichloracetic acid is one of a group of similar acids; there are two others in the group, the monochlor and the dichloracetic acids. I never have seen the other two, but they have similar properties, and the slight modification in their composition, due to the difference in their respective proportions of chlorin, might make some difference in the combination that would be of value in dental therapeusis.—*Dental Cosmos.*

